# Supplementary material for: Type VI secretion system MIX‐effectors carry both antibacterial and anti‐eukaryotic activities
Source: EMBO Rep. 2017 Sep 14;18(11):1978–90. doi: 10.15252/embr.201744226 (PMC5666596; doi:10.15252/embr.201744226)
Supplement: Supplementary file 1 — Appendix [file EMBR-18-1978-s001.pdf]

Appendix for:

Type VI secretion system MIX-effectors are targeted at both bacterial and eukaryotic neighbors

Table of Contents

Appendix Figure S1. T6SS1 is most active under warm marine-like conditions. 2

Appendix Figure S2. Exogenous complementation of VgrG1 does not rescue loss of bactericidal activity. 3

Appendix Figure S3. Deletion of tssG1 does not affect Vpr growth. 4

Appendix Figure S4. Schematic representation of the gene neighborhoods of new Vpr T6SS1 effectors identified by comparative proteomics. 5

Appendix Figure S5. Vpr01570 contains a C-terminal CNF toxin domain. 6

Appendix Figure S6. Vpr01s_11_00400 is homologous to C-terminus of insecticidal toxin Txp40. 7

Appendix Figure S7. Deletion of vprh does not impair Vpr bactericidal activity. 8

Appendix Figure S8. Deletion of vpr01570 does not affect T6SS1 activity or bacterial growth. 9

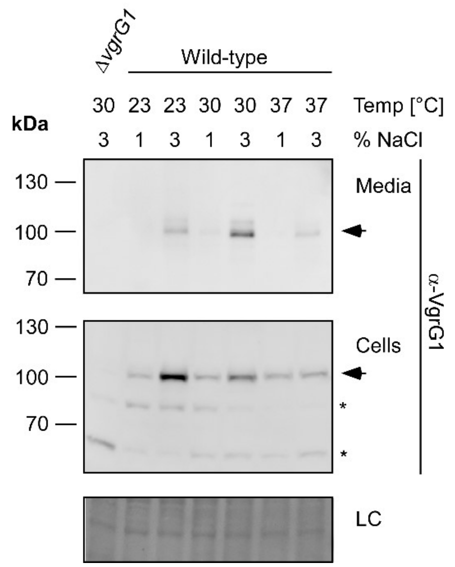

Appendix Figure S1. T6SS1 is most active under warm marine-like conditions. Expression (Cells) and secretion (Media) of *Vpr* VgrG1 after 5 h incubation under the indicated temperature and salinity conditions. Δ*vgrG1* deletion strain was used as negative control. VgrG1 was detected by immunoblot using specific antibodies against VgrG1. Arrow marks VgrG1 signal; asterisks mark non-specific bands. Loading control (LC) is shown for total protein lysate. Experiment was performed twice with similar results. A representative experiment is shown.

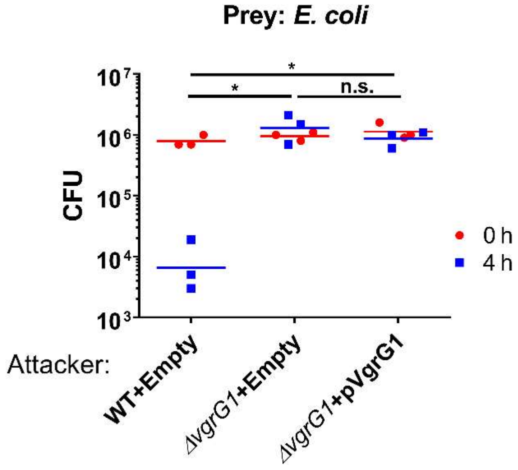

Appendix Figure S2. Exogenous complementation of VgrG1 does not rescue loss of bactericidal activity. Viability counts of *E. coli* prey before (0 h) and after (4 h) co-culture with indicated *Vpr* strains on media containing 3% NaCl at 30°C. Attackers contain either an empty expression vector (Empty), or vector for arabinose-inducible expression of VgrG1. Asterisks mark statistical significance between samples at 4 h timepoint ($P<0.05$) determined using unpaired, two-tailed Student *t* test; n.s.=not significant; WT=wild-type.

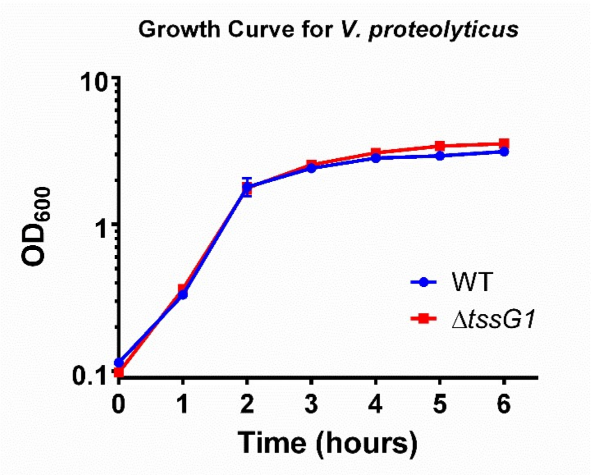

Appendix Figure S3. Deletion of *tssG1* does not affect *Vpr* growth. Growth of *Vpr* strains in MLB (3% NaCl) at 30°C measured as absorbance optical density at 600 nm (OD_{600}). Data are means ± standard deviation (n=3). WT=wild-type.

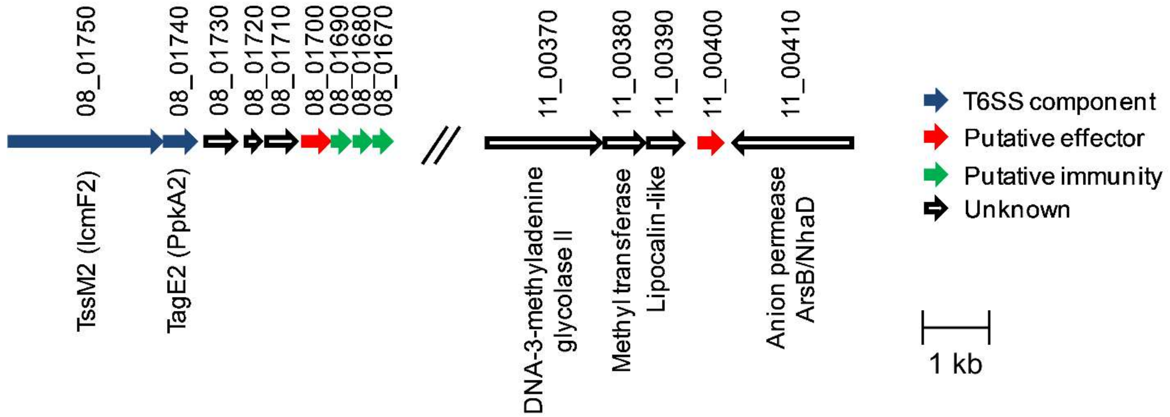

Appendix Figure S4. Schematic representation of the gene neighborhoods of new *Vpr* T6SS1 effectors identified by comparative proteomics. Genes are represented by arrows indicating direction of translation. Locus tags (*vpr01s_xx_xxxxx*) shown above. Encoded proteins and known domains shown below.

```
Vpr01570  313 MVGEWDGEGYSTGEQVSMTQLTGRGPFYLKTPRAPRAKFWFSRIDQRAEFSAEKASERLS
VopC       88 NVKKADAKSITALKSGDLHILKGKGIIGMKG------------GDNKLPFKCTIVNDD--
CNF1      720 SIESTSKSNFQKLSRGNIDVLKGRGSISSTRQRAIYPYFEAANADEQQPLFFYIKKDRFD
CNFy      720 NVAEISSINFRRLNSGNINVLKGRGVFSSRRLREIYLRFDAANADELRPGDVYVKKTKFD

Vpr01570  373 DSDQRDVLECSDGTQMTSFNKSMNAWV---------------THFWDDNPLPEELDVLVL
VopC      134 -KNG-AHLSQGTNLA-TNG---IKSMAGDSVRAAQLIPGTPLGQFYNSAPLDDSFNVVHL
CNF1      780 -NHGYDQYFYDNTVG-LNGIPTLNTYTGEIPSDSSSLG----STYWKKYNLTNETSIIRV
CNFy      780 -SMGYDSHFYNEGIG-INGAPTLNTYTGEYVADSSSQG----ATYWLKYNLTNETSIIKV

                                              *              *
Vpr01570  418 HNGYTGAAGVRIDLDELDPGQQILVTGGTLSGCTMVTGVKGRQFYALHAGT-AI PNEWK
VopC      188 PNGQRGVNGLKIPLSEFYSEKKFLFSNGALSGCMTCTAIDKNNLYIFHVGKDGNDTSPWK
CNF1      834 SNSARGANGIKIALEEVQEGKPVIITSGNLSGCTTIVARKEGYIYKVHTGT-TK  A-GF
CNFy      834 SNSARGANGIKIALEEIEENKPVVITSGTLTGCTVVFARKGEYFYAVHTGN-SESLI-GF

Vpr01570  477 TGQHGVRDNYALLKLMMPENDLTAPPEWDNDGMPALL-DCFSQATMAYSG---KPEAKMQ
VopC      248 TNVDGSSLIQKNMKMLLGQNSDSLNNGIQ--GLIDYCSKNFDKAIIQYCGHGEQYSG---
CNF1      892 TSTTGVKKAVEVLELLTKEPIPRVEGIMSNDFLVDYLSENFEDSLITYSSSEKKPDSQIT
CNFy      892 TSTSGVAKAIEVLSSLSELEVPALPDVINNNTLVEYLSDNFDSALISYSSSSLKPNSMIN

Vpr01570  532 --RADARCF
VopC      303 --RKNIHLF
CNF1      952 IIRDNVSVF
CNFy      952 ISRENVSTF
```

Appendix Figure S5. Vpr01570 contains a C-terminal CNF toxin domain. Multiple sequence alignment of Vpr01570 MIX-effector C-terminal toxin domain and other CNF toxin domains. Residue numbers shown left of the alignment. Conserved residues marked in yellow (similar) and red (identical). Conserved catalytic residues marked by asterisk. Alignments shown for: Vpr01570 (GAD68163), *V. parahaemolyticus* VopC (VPA1321), *E. coli* CNF1 (UTI89_C4921), and *Yersinia pseudotuberculosis* CNFy (YPK_2615).

```
VPR01S_11_00400     1 M-----------------------------------------------------------
V. mimicus          1 M-----------------------------------------------------------
Txp40_Xenorhab.     1 MVENTVVENTMVIKPVTTPSVIQLTPDDRGEYQPVEKQIAGDIIRVLEFKQTNESHTGLY
Txp40_Photorhab.    1 M-------------------VIQLTPDDRSGYPPVEKQIAGDIVRILNFKQTDEGHTASY

VPR01S_11_00400     2 ------------------------------------------------------------
V. mimicus          2 ------------------------------------------------------------
Txp40_Xenorhab.    61 GIAYRAKKVIIAYALAVSGIHNVSQLPEDYYKNKDNTGRIYQEYMSNLSSALLGENGDQI
Txp40_Photorhab.   42 GIEYRAKKIILAYALAVSGIHNVSKLPDDYYKNKETAERIYQEYMSNLSSALLGENGDQI

VPR01S_11_00400     2 ------------------------------------------------------------
V. mimicus          2 ------------------------------------------------------------
Txp40_Xenorhab.   121 SKDMANDFTQNELEFGGQRLKNTWDIPDLENKLLEDYSDEDKLLALYFFASQELPMEANQ
Txp40_Photorhab.  102 SKDMANGFYKNELDFEGQYPQNIWNVPELENKPLSAYSDDDKLLALYFFSVQEIPLEENQ

VPR01S_11_00400     2 ------------------------------------------------------------
V. mimicus          2 ------------------------------------------------------------
Txp40_Xenorhab.   181 QSNAANFFKVIDFLLILSAVTSLGKRIFSKNFYNGLETKSLENYIERKKLSKPFFRPPQK
Txp40_Photorhab.  162 QSNAARFFKLIDFLLTLSAVTSLGRRIFSKNFYNGLESKSLENYIERKKFPKPFFRPPQR

VPR01S_11_00400     2 ---------------SH-----------------WRINKSAVRW------SRPISSAAQR
V. mimicus          2 ---------------PG-----------------WRLDKSRVNW------NRTITSAAQD
Txp40_Xenorhab.   241 LPDGRTGYLAGPPKAPKLPTTSSTATTSTAASSNWRVSLQKLRDNPSRNAFMQMDDAAKR
Txp40_Photorhab.  222 LPDGRIGYLAGPTEAPK-----------------WRVSFKELKNNKSRNGFSNMEGAAKQ

VPR01S_11_00400    24 NFDLFQNEIFHNGTDPRHAADEFDS----HVECLDKGNNVWSIRLSQRDRVVFRLDETDH
V. mimicus         24 KLDMFQNEIFNKGTDPRIAAKQFDS----HCECLDRTNNIWSIRLSQSDRVVFRINDIDH
Txp40_Xenorhab.   301 KYSSFIKEV-QKGNDPRAAATSIGTKSGSNFEKL-QGRDLYSIRLSQEHRVTFSINNTDQ
Txp40_Photorhab.  265 KYSSFIKEV-QKGNAPQTAAKSIGTASGSNLEKL--PNNLYSVRLSQKDRVTFTQNDTDN

VPR01S_11_00400    80 VCIIEGVGGHY---
V. mimicus         80 ICVIEAVGGHY---
Txp40_Xenorhab.   359 IMEIQSVGTHYQNI
Txp40_Photorhab.  322 TMTVHSVGTHYKNI
```

Appendix Figure S6. Vpr01s_11_00400 is homologous to C-terminus of insecticidal toxin Txp40. Multiple sequence alignment of Vpr01s_11_00400 and Txp40 toxins. Residue numbers shown left of the alignment. Conserved residues marked in yellow (similar) and red (identical). Alignments shown for: Vpr01s_11_00400 (GAD68047), AL543_05135 from *Vibrio mimicus* (AMG02395), Txp40 from *Xenorhabdus nematophila* AN6/1 (CEK22105), and Txp40 from *Photorhabdus luminescens* subsp. *laumondii* (KTL63163).

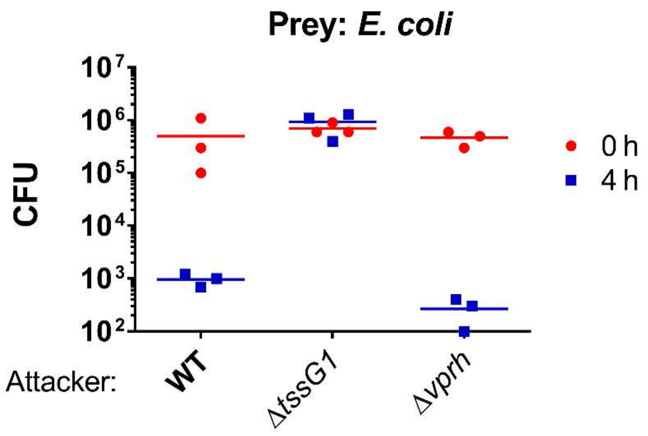

Appendix Figure S7. Deletion of *vprh* does not impair *Vpr* bactericidal activity. Viability counts of *E. coli* prey before (0 h) and after (4 h) co-culture with indicated *Vpr* strains on media containing 3% NaCl at 30°C. WT=wild-type.

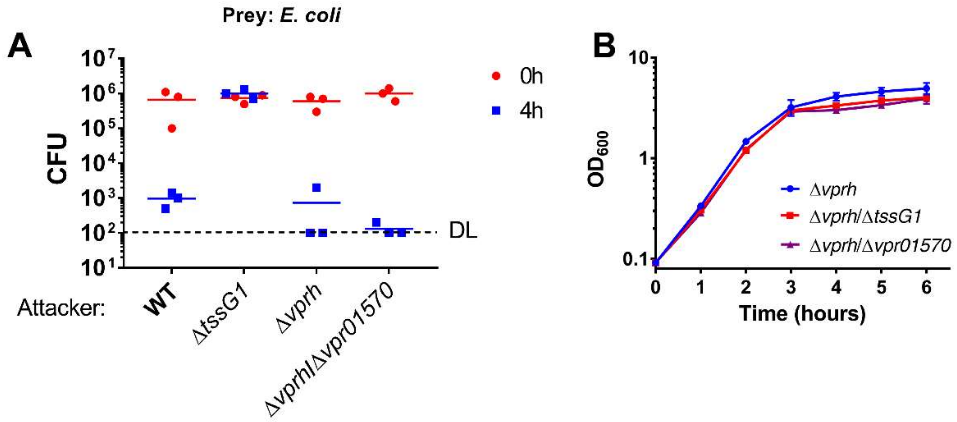

Appendix Figure S8. Deletion of *vpr01570* does not affect T6SS1 activity or bacterial growth. (**A**) Viability counts of *E. coli* prey before (0 h) and after (4 h) co-culture with indicated *Vpr* strains on media containing 3% NaCl at 30°C. WT=wild-type; DL=detection limit. (**B**) Growth of *Vpr* strains in MLB (3% NaCl) at 30°C measured as absorbance optical density at 600 nm (OD_{600}). Data are means ± standard deviation (n=3). WT=wild-type.
