# Supplementary material for: Type VI secretion system MIX‐effectors carry both antibacterial and anti‐eukaryotic activities
Source: EMBO Rep. 2017 Sep 14;18(11):1978–90. doi: 10.15252/embr.201744226 (PMC5666596; doi:10.15252/embr.201744226)
Supplement: Supplementary file 2 — Expanded View Figures PDF [file EMBR-18-1978-s002.pdf]

Expanded View Figures

Figure EV1. Vpr01570 is a functional anti-eukaryotic toxin.

A Confocal micrograph of HeLa cells transfected with vectors expressing the indicated proteins. Cells were stained for F-actin and DNA using rhodamine-phalloidin (red) and Hoechst stain (blue), respectively. Scale bar = 30 μm; sfGFP, superfolder green fluorescent protein. Arrowheads mark actin ruffles.

B Growth of BY4741 yeast containing vectors for galactose-inducible expression of indicated proteins on repressing (glucose) and inducing (galactose) plates. Yeast were spotted in 10-fold serial dilutions. $VopR^{\Delta 90/CA}$ is a truncated and catalytically inactive form of the *Vibrio parahaemolyticus* type III effector VopR and serves as a non-toxic control.

C, D Immunoblot using anti-GFP (C) or anti-Myc (D) antibodies to verify expression of proteins used in (A) and (B), respectively. In (C), black arrow marks expected size of Vpr01570-sfGFP fusions and white arrow marks expected size of sfGFP. In (D), black arrow marks the expected size of Vpr01570-myc, and white arrow marks the expected size of $VopR^{\Delta 90/CA}$-eGFP fusion. LC, loading control.

Source data are available online for this figure.

▶

© 2017 The Authors

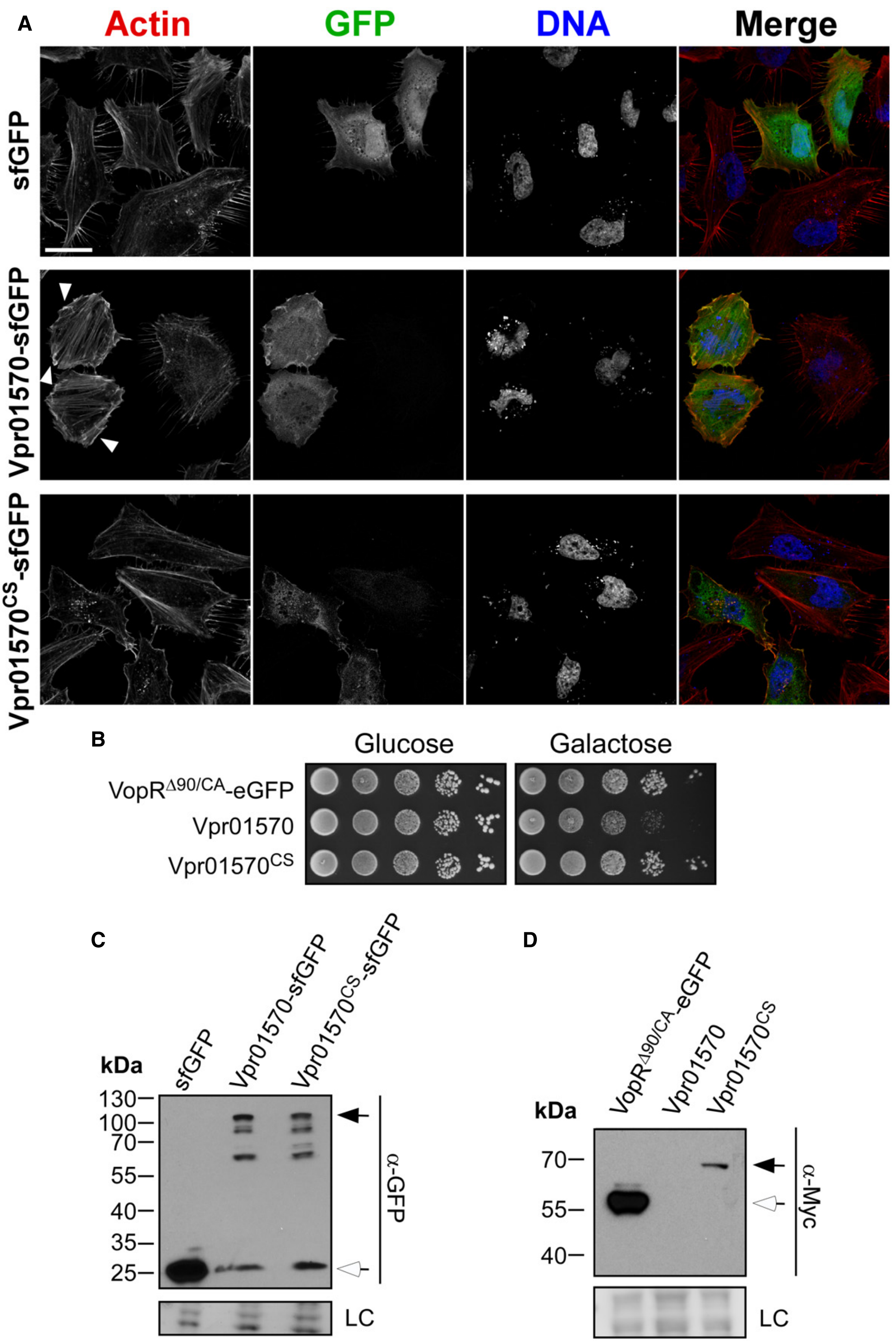

Figure EV1.

 © 2017 The Authors

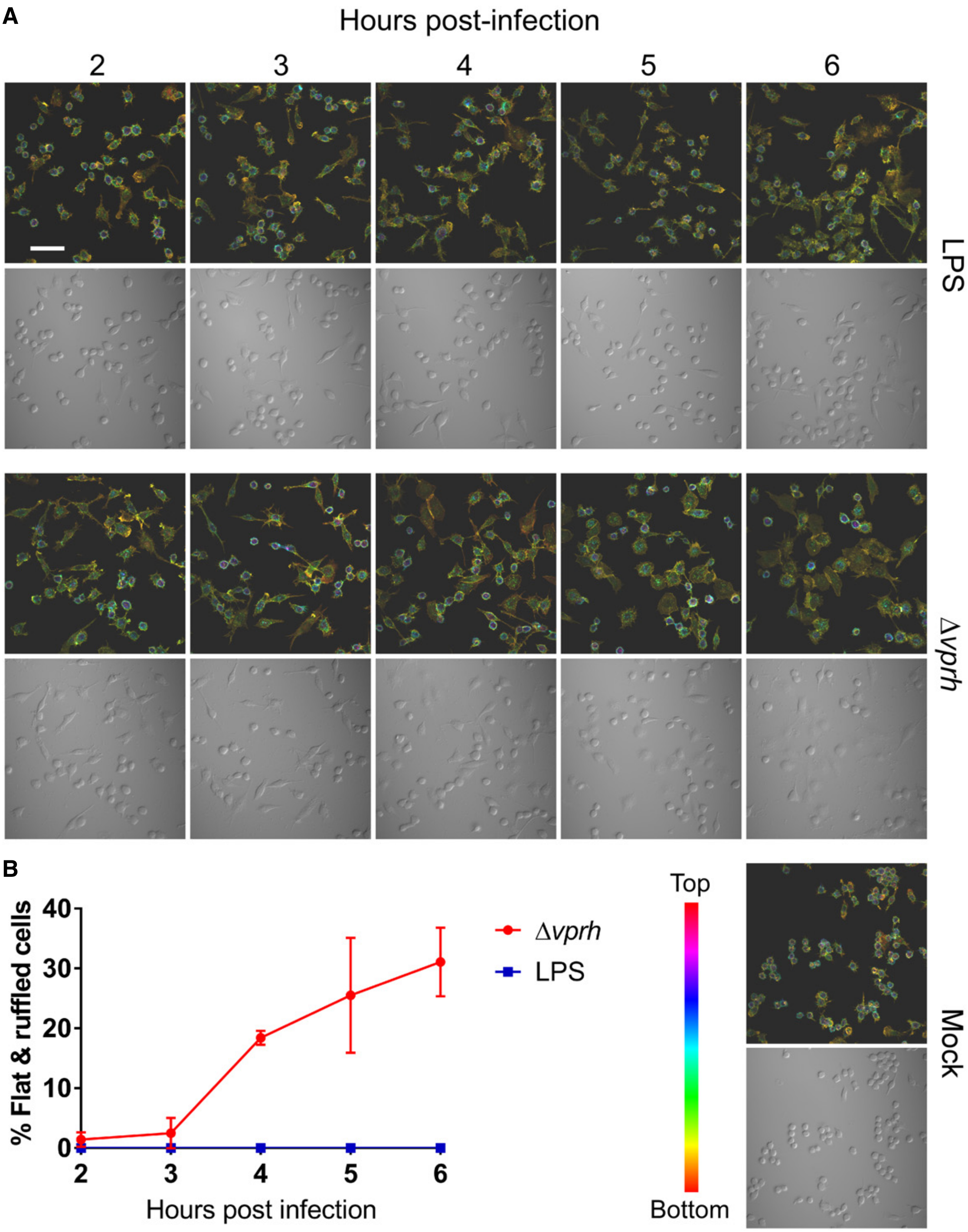

Figure EV2. ***Vpr*-induced morphological changes in macrophages begin 4 h post-infection.**

A Confocal micrograph of RAW 264.7 cells infected with *Vpr* Δ*vprh* strain at MOI = 25 or treated with LPS (0.1 μg/ml) for the indicated times. Cells were stained for F-actin using rhodamine-phalloidin. Top panels show fluorescence images. F-actin is color-coded to show cell depth in the *z*-axis. Bottom panels show DIC of same fields of view. Scale bar = 50 μm.

B Quantification of percentage of infected cells presenting a flatten and ruffled morphology during *Vpr* infection or LPS treatment as in (A). Results shown as mean percentage of cells in a field ± standard deviation (*n* = 3, minimum number of cells per field is 41; maximum number of cells per field is 95). Experiment was performed three times, and a representative result is shown.

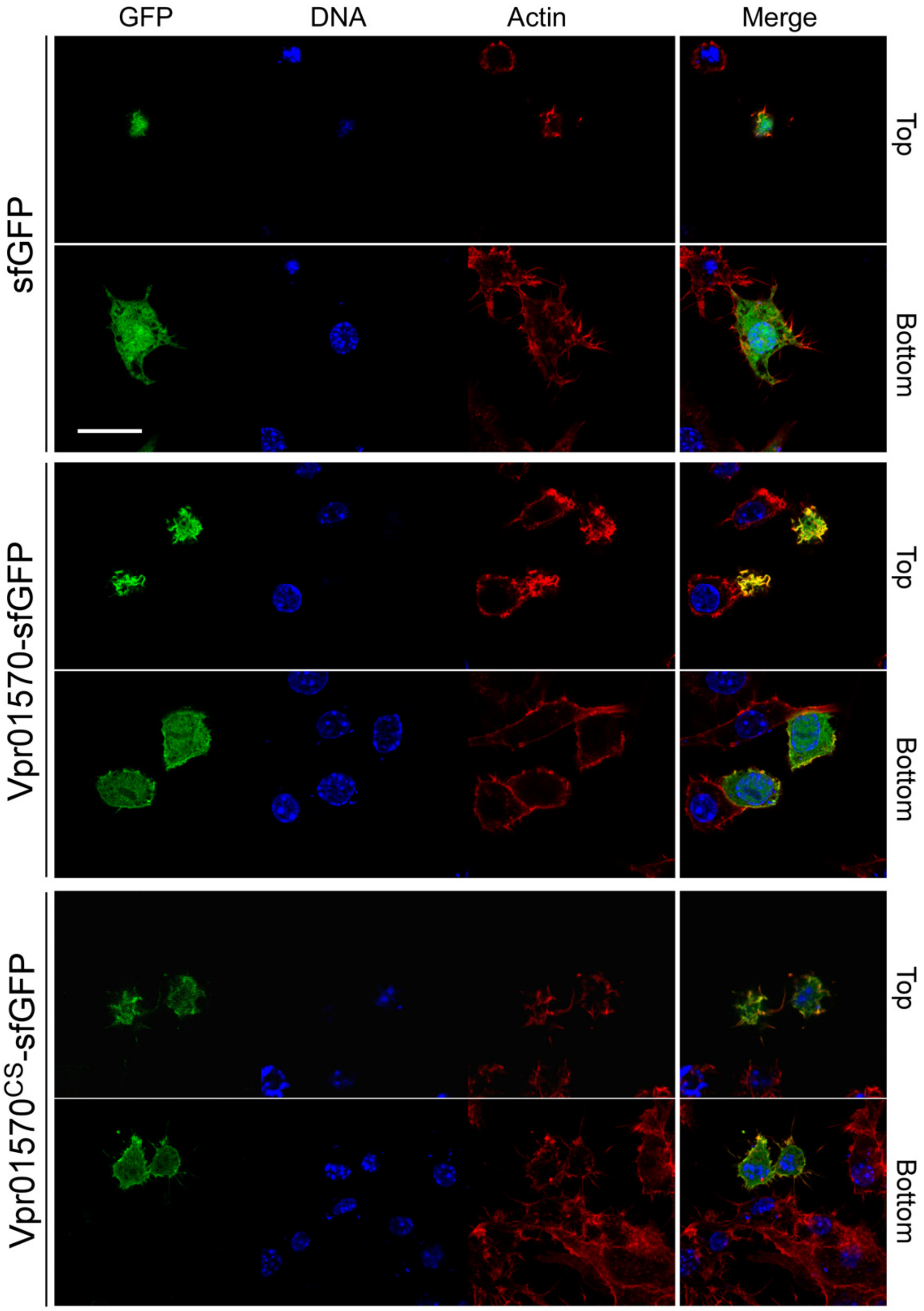

Figure EV3. Vpr01570 is sufficient to induce actin ruffles in macrophages.

Confocal micrograph of RAW 264.7 cells transfected with vectors expressing the indicated proteins. Cells were stained for F-actin and DNA using rhodamine-phalloidin (red) and Hoechst stain (blue), respectively. Top panels show a focal plane at the top of the cells; bottom panels show a focal plane located at the bottom of the cells. Scale bar = 20 μm; sfGFP, superfolder green fluorescent protein.

 © 2017 The Authors
