# Supplementary figures and images for: Type VI secretion system MIX‐effectors carry both antibacterial and anti‐eukaryotic activities
Source: EMBO Rep. 2017 Sep 14;18(11):1978–90. doi: 10.15252/embr.201744226 (PMC5666596; doi:10.15252/embr.201744226)

## Appendix Figure S1

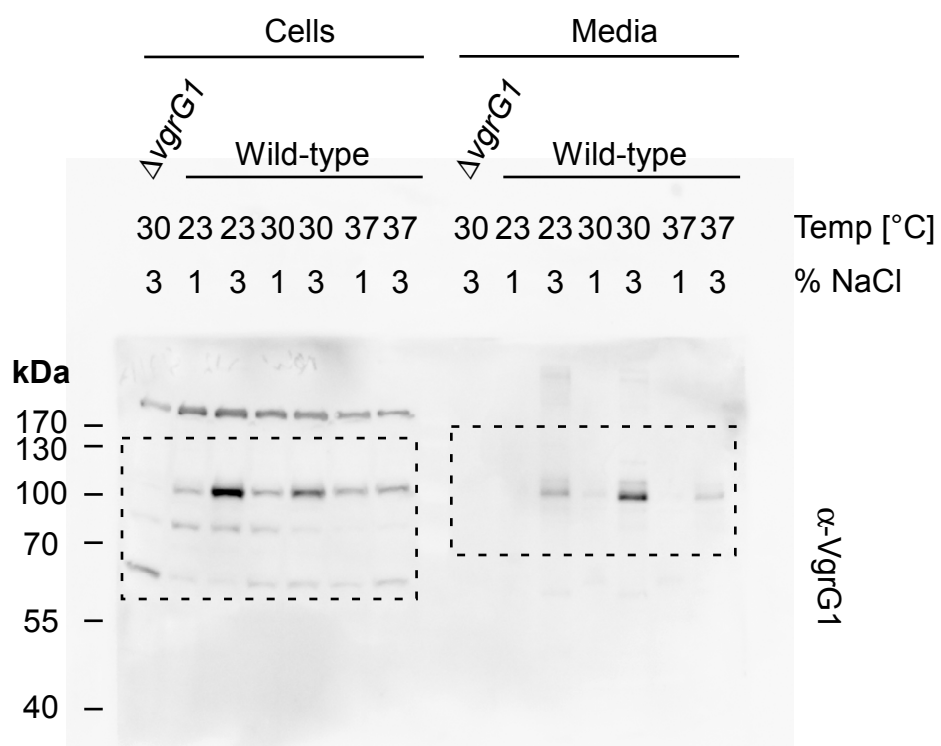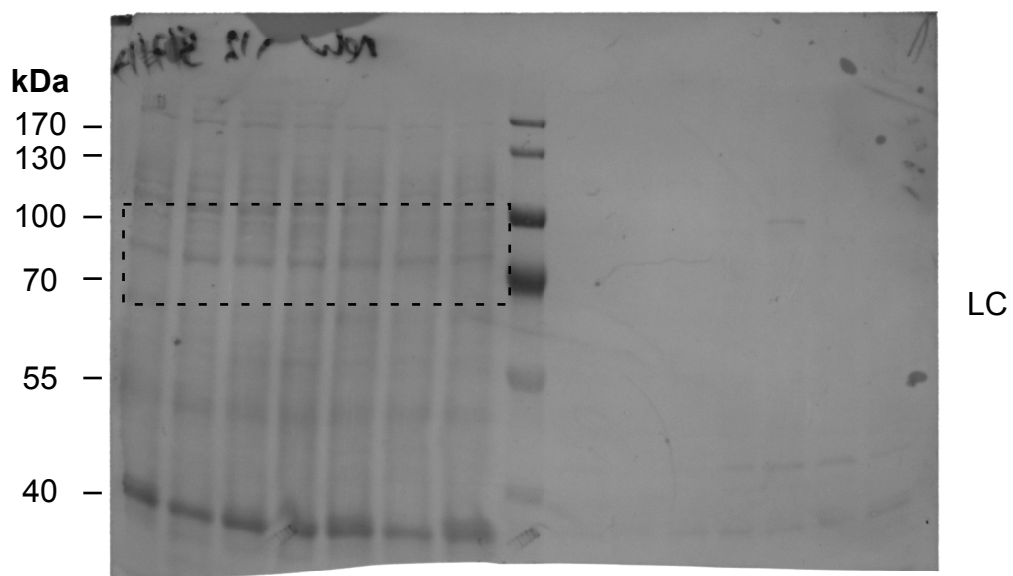

Supplement: Supplementary file 4 — Source Data for Expanded View and Appendix [file EMBR-18-1978-s005.zip › SourceData_AppendixFigS1/EMBOR-2017-44226V2_SDataAppendixFigS1.pdf]

Figure EV1 C

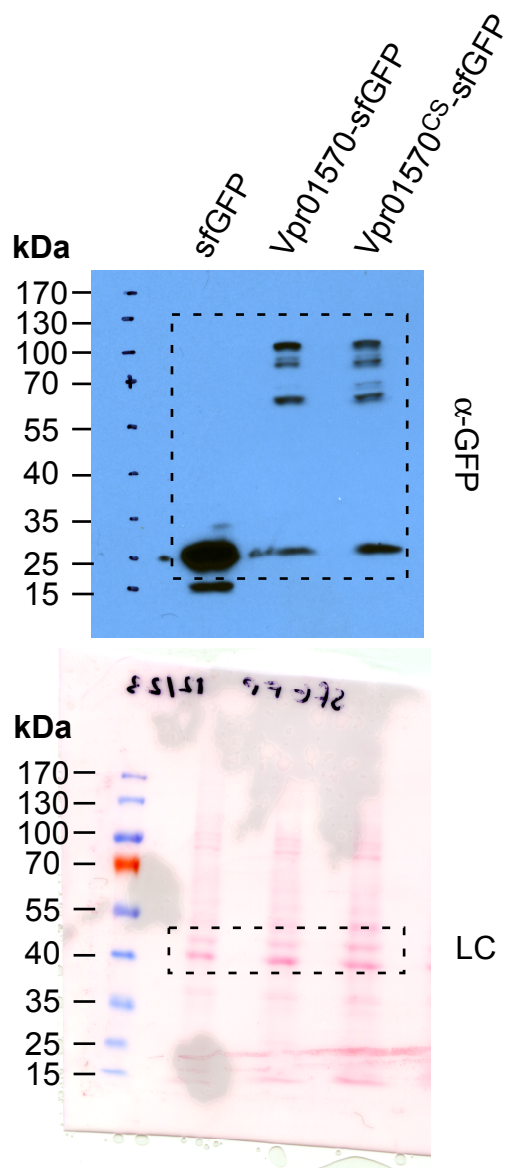

Figure EV1 D

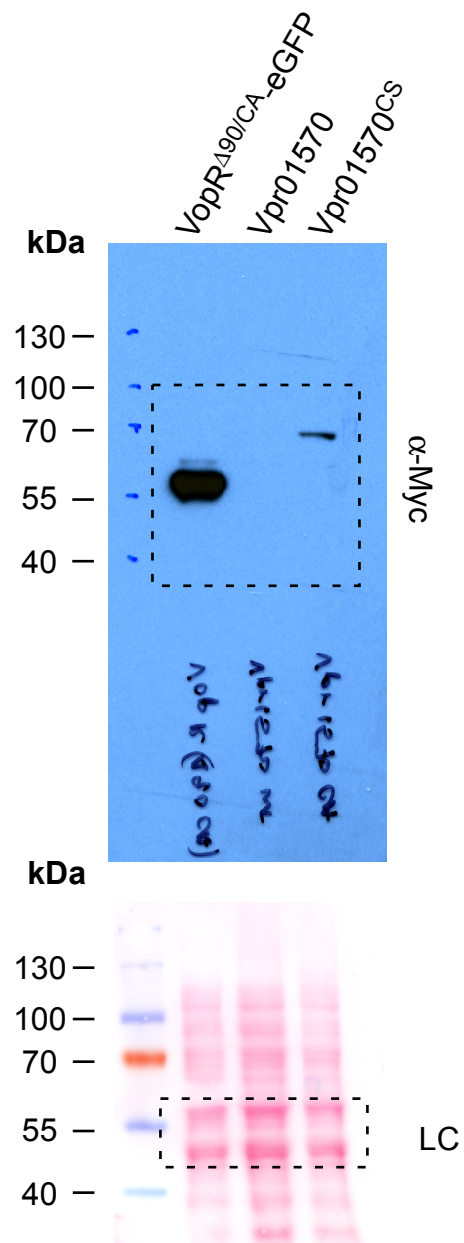

Supplement: Supplementary file 4 — Source Data for Expanded View and Appendix [file EMBR-18-1978-s005.zip › SourceData_FigEV1/EMBOR-2017-44226V2_SDataFigEV1.pdf]

**Figure 3D**

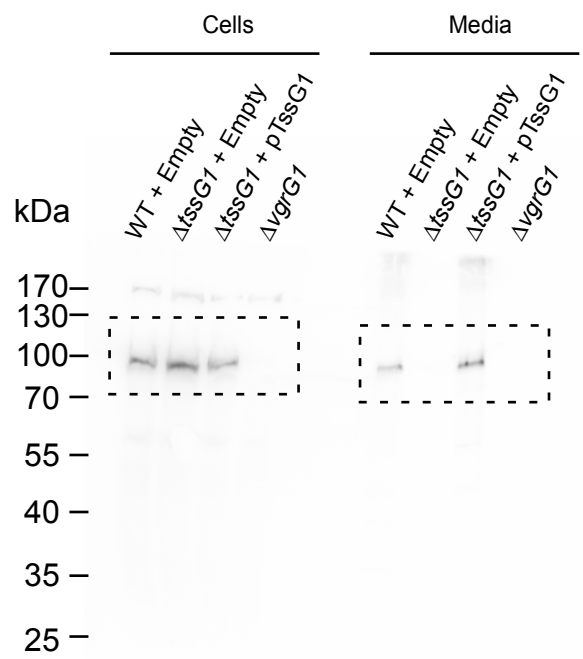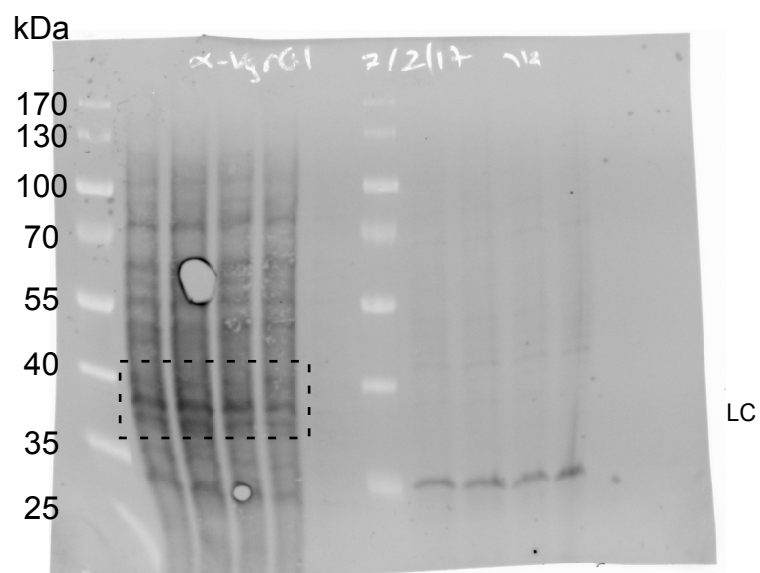

Images were acquired using a Vilber Lourmat Fusion FX-6 imager

Supplement: Supplementary file 6 — Source Data for Figure 3D [file EMBR-18-1978-s004.pdf]
